# Molecular Biology in Pediatric High-Grade Glioma: Impact on Prognosis and Treatment

**DOI:** 10.1155/2015/215135

**Published:** 2015-09-13

**Authors:** Daniela Rizzo, Antonio Ruggiero, Maurizio Martini, Valentina Rizzo, Palma Maurizi, Riccardo Riccardi

**Affiliations:** ^1^Department of Pediatric Oncology, A. Gemelli Hospital, Catholic University of Rome, Largo A Gemelli, 1, 00168 Rome, Italy; ^2^Anatomic Pathology, Catholic University, “A. Gemelli” Hospital, 00168 Rome, Italy

## Abstract

High-grade gliomas are the main cause of death in children with brain tumours. Despite recent advances in cancer therapy, their prognosis remains poor and the treatment is still challenging. To date, surgery followed by radiotherapy and temozolomide is the standard therapy. However, increasing knowledge of glioma biology is starting to impact drug development towards targeted therapies. The identification of agents directed against molecular targets aims at going beyond the traditional therapeutic approach in order to develop a personalized therapy and improve the outcome of pediatric high-grade gliomas. In this paper, we critically review the literature regarding the genetic abnormalities implicated in the pathogenesis of pediatric malignant gliomas and the current development of molecularly targeted therapies. In particular, we analyse the impact of molecular biology on the prognosis and treatment of pediatric high-grade glioma, comparing it to that of adult gliomas.

## 1. Introduction

Brain tumors are the most common solid tumors affecting childhood and the main cause of cancer-related death in children. Gliomas make up approximately 60% of all pediatric brain tumors and about half of these are considered high-grade malignancies. In particular, pediatric glioblastoma (GBM, grade IV WHO) accounts for 15% of all pediatric brain tumours [1]. Despite efforts to improve treatment, children with high-grade glioma (HGG) still have a dismal outcome with a 5-year survival of less than 20% [2].

Surgery followed by radiotherapy (RT) is the standard treatment of patients with HGG. Surgery has prognostic significance in patients with near total resection and at present it is the strongest indicator of prognosis in pediatric HGG. Focal RT is used as first-line therapy except in children under 3 years. Recently, the combination of RT and temozolomide (TMZ) followed by adjuvant TMZ showed a superior outcome compared to RT in the treatment of adults with newly diagnosed GBM [3]. However, several studies on the use of TMZ in the treatment of children with HGG were performed with disappointing results (Table 1) [4–9].

**Figure 1:**
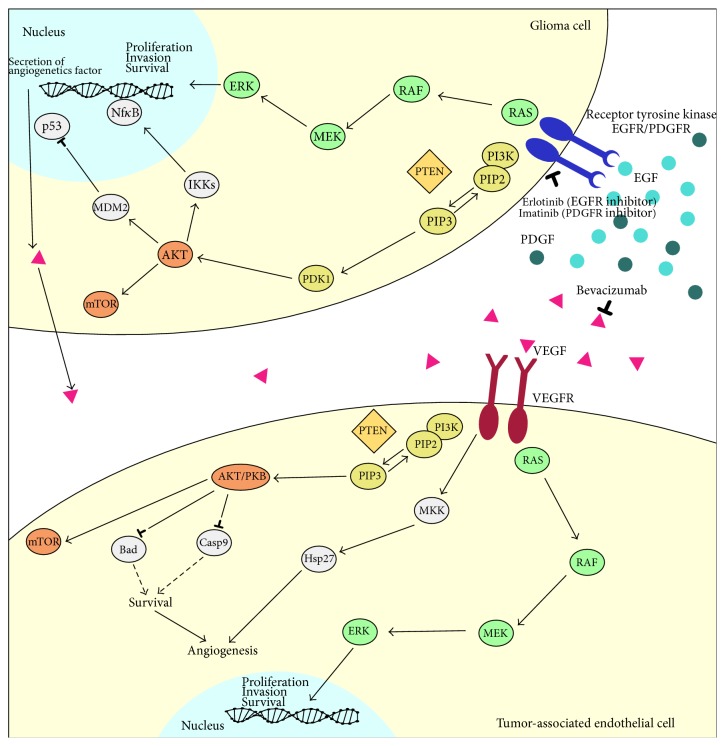
Molecularly targeted therapy for pediatric malignant glioma.

Recognizing the limitations of current standard therapy for HGG, over the past few years substantial advances have been made in molecular biology in identifying new therapeutic approaches. Molecular biological investigations have confirmed that the transformed phenotype of HGG is highly complex and is the result of the dysfunction of a variety of interrelated regulatory pathways (Figure 1) [10]. HGG displays complex chromosomal and genetic alterations leading to the inactivation of various tumor suppressor genes, as well as aberrant activation of protooncogenes. The molecular profiling of HGG in adults has been studied intensively because of its relatively high incidence in this population. Conversely, relatively few studies have been performed on pediatric HGG due to the difficulty in obtaining a large enough series of patient samples. Significant differences exist between adult and pediatric HGG, suggesting that the pathways and mechanisms of malignant gliomagenesis are molecularly distinct [11, 12]. In this review the genetic abnormalities known to be implicated in the pathogenesis of pediatric HGG are described, underlining their prognostic and predictive role and their main impact on the treatment, including the results achieved with new therapeutic agents directed against rational molecular targets (Table 1).

## 2. O6-Methylguanine-DNA Methyltransferase (MGMT)

The antitumor activity of TMZ is due to DNA methylation and O6-methylguanine-DNA methyltransferase (MGMT) is one of the suggested mechanisms of chemoresistance [13]. Cytotoxicity of TMZ is initiated by the methylation of the O6 position of guanine which causes mispairing of O6-methylguanine with thymine. The futile repair of this base mismatch by the mismatch repair system generates single- and double-strand DNA breaks that activate cell death. MGMT prevents this process by transferring methylating groups from the O6 position of guanine to one of its internal cysteine residues, neutralizing the cytotoxic effect of alkylating agents. Therefore elevated MGMT activity is associated with enhanced resistance to alkylating agents.

Stupp et al. showed that* MGMT* promoter methylation was associated with loss of MGMT expression and diminished DNA-repair activity. Therefore,* MGMT* promoter methylation represents an independent favorable prognostic factor for GBM patients and a predictive marker of benefit from alkylating agent therapy in GBM [3]. From literature, the percentage of the methylated* MGMT* promoter of adults ranges from 24% to 68%, averaging about 40% [14–16].

Most studies on* MGMT* methylation in HGG have been conducted in adults, probably due to the relatively rare incidence in children. Only few studies in pediatric populations have been reported [17–20], showing a similar methylation status in children and adults and significant correlation between the methylation status and clinical outcome. In particular, a retrospective study of 10 pediatric patients with GBM showed* MGMT* promoter methylation in 4 of 10 patients and revealed a significant association between* MGMT* promoter methylation and prolonged survival (*P* = 0.01) [18]. The average survival time for patients with methylated* MGMT* was 13.7 months as compared to 2.5 months for the patients with unmethylated* MGMT* promoter (*P* = 0.01). Moreover, the patients receiving TMZ that had the methylated* MGMT* gene promoter responded better to treatment (*P* = 0.01) [18]. In a further study DNA methylation was also associated with increased median event-free survival (EFS) and overall survival (OS) in children with relapsed HGG (5.5 months versus 0.9 months, *P* = 0.015) [21].

Unlike previous studies, recently Lee et al. showed that the incidence of methylation of the* MGMT* promoter in pediatric GBM is rare [22]. In this study the methylation status was assessed using two methods: conventional methylation-specific polymerase chain reaction and, for the first time in pediatric patients, methylation-specific multiplex ligation-dependent probe amplification. Both methods showed a surprisingly low proportion of methylated samples (6% and 16%, resp.). Moreover, there was no difference between the methylated and unmethylated groups in either progression-free survival (PFS) or OS. Based on limitations of this study, such as small number of patients, heterogeneity in terms of adjuvant treatment modality, location of tumor, and extent of resection, the results should be taken with caution and a multicenter collaborative trial with a larger number of patients should be performed.

## 3. Epidermal Growth Factor Receptor (EGFR)

Epidermal growth factor receptor (EGFR) is a membrane-anchored protein tyrosine kinase that, when phosphorylated, activates a variety of downstream effector molecules regulating cell proliferation and differentiation (Figure 1).

Aberrant cell signaling via the EGFR family has been implicated in the development of several human cancers, including brain tumors. Moreover, recent research has shown a pivotal relationship between EGFR overexpression or* EGFR* amplification and disease progression, poor survival, resistance to chemotherapy, and poor response [23].* EGFR* amplification and EGFR overexpression affect 30–50% of adult GBM [24]. In pediatric HGGs available data suggest that* EGFR* amplification occurs with low frequency, averaging about 3% [11, 25], although in a recent study Bax et al. found a greater prevalence of* EGFR* gene amplification and* EGFRvIII* mutation in pediatric HGG than had previously been recognized [26].* EGFRvIII* is the most common mutant of* EGFR* gene reported in GBM. The ability of this variant to “switch on” cell signaling without ligand stimulation, even though it does not dimerize, plays an important role in cancer pathogenesis.

Erlotinib, a small-molecule EGFR inhibitor, has been shown to inhibit EGFRvIII by blocking constitutive EGFRvIII kinase activity and the growth of EGFRvIII transformed cells [27, 28]. However, several clinical trials have demonstrated a limited activity of erlotinib in the treatment of adults and children with recurrent HGG [29–33]. In particular only a subset of patients treated by EGFR inhibitors showed a response to these agents. Mellinghoff et al. demonstrated that coexpression of EGFRvIII and phosphatase and tensin homolog (PTEN) was associated with better response in patients with HGG treated by EGFR inhibitors [27]. Haas-Kogan et al. showed that patients with GBM whose tumors expressed high levels of EGFR and low levels of phosphorylated protein kinase B/Akt responded better to erlotinib therapy [34]. A recent study indicated that 5 genes within the* EGFR* signaling pathway (*STAT1*,* FKBP14*,* RAC1*,* PTGER4*, and* MYC*) may modulate the response of adult GBM to erlotinib [35]. In the study by Bax et al. phosphorylated receptor tyrosine kinase profiling showed a specific activation of platelet-derived growth factor receptor (PDGFR) *α*/*β* in EGFRvIII-transduced pediatric GBM cells and targeted coinhibition with erlotinib and imatinib, an inhibitor of the tyrosine kinases Bcr-Abl, Kit, and PDGFR, could lead to enhanced efficacy [26]. This suggests that the erlotinib-associated signaling pathway is a complex one and needs to be taken into account in future trials.

## 4. Platelet-Derived Growth Factor Receptor (PDGFR)

Platelet-derived growth factor (PDGF) is a major regulator of angiogenesis [36] and is involved in the regulation of proliferation, neuronal differentiation, and motility of cells within the nervous system [37]. PDGF encompasses a family of ligands (AA, AB, BB, CC, and DD) that bind to a pair of receptors (PDGFR*α* and PDGFR*β*) [38]. Concurrent expression of one or more of these ligands and their receptors has been observed in a high percentage of malignant gliomas [38, 39], thus allowing autocrine and paracrine stimulation [38, 40]. In particular, PDGFRA is the most frequent target of focal amplification in pediatric HGGs arising within and outside the brainstem [11, 41–44] and somatic mutations of* PDGFRA* have been recently reported in pediatric HGGs [45, 46]. In contrast, EGFR is the predominant receptor tyrosine kinase targeted by both amplification and mutation in adult GBM [25, 47].

Amplification of wild-type* PDGFRA* occurred more frequently in tumors within the brainstem (26% DIPG versus 11% nonbrainstem HGG, *P* = 0.04), whereas* PDGFRA* sequence mutations were more common in pediatric HGG arising outside the brainstem, although this was not statistically significant (14% nonbrainstem HGG versus 5% DIPG, *P* = 0.14 [11, 41]).

Imatinib (Gleevec) is a molecular targeted drug which selectively inhibits several receptor tyrosine kinases, including PDGFR (Figure 1). Imatinib was evaluated in several clinical trials, with the drug showing limited activity in the monotherapy of adult GBM [48, 49]. Conversely, a substantial antitumor activity of imatinib in combination with hydroxyurea was described in further open-label trials [50]. Nevertheless, the results have been disappointing in pediatric HGG [51] (Table 1) and further trials looking at combination therapy are required. The main reason for the limited activity of imatinib may be that inhibition of PDGFR alone is insufficient to prevent growth of malignant gliomas. Signaling through the Ras-mitogen-activated protein kinase and Akt pathways as a result of* EGFR* amplification and mutation and deletion of* PTEN*, respectively, may result in tumor growth even in the presence of PDGFR inhibition.

## 5. Phosphatase and Tensin Homolog (PTEN)/Akt

The phosphatidylinositol 3-kinase (PI3K) pathway is involved in several important cellular functions, including growth control, survival, and migration. Following its activation by growth factor receptors, including EGFR, EGFRvIII, and PDGFR, PI3K catalyzes the addition of a phosphate to phosphatidylinositol-4,5,-bisphosphate to form phosphatidylinositol-3,4,5,-triphosphate (PIP3), which initiates many of its tumorigenic activities via Akt (Figure 1). Akt is recruited to the plasma membrane by PI3K mediated formation of PIP3, leading to Akt phosphorylation at thr308 and Ser473 (via phosphoinositide-dependent kinase-1 and phosphoinositide-dependent kinase-2, resp.). Akt phosphorylation at these 2 sites activates its kinase function, leading to downstream signaling promoting proliferation and inhibiting apoptosis.

The* PTEN* gene is an important regulator of the PI3K pathway (Figure 1). It is a tumor suppressor gene which encodes a phosphatase catalyzing the dephosphorylation of PIP3, thus inhibiting activation of the Akt pathway. When* PTEN* is altered, the Akt pathway can become constitutively active. Elevated Akt levels have been associated with loss of PTEN in many GBMs. Moreover, in glial tumors,* PTEN* mutation frequency increases with increasing tumor grade and is associated with poorer outcome [52, 53].

Although* PTEN* mutations are found in approximately 50% of adult HGG, predominantly GBM, a relatively small rate of mutation was found in childhood gliomas [25, 53, 54]. However, in a recent study Pollack et al. observed that activation of Akt is a common finding in pediatric malignant gliomas. In particular, 42 (79%) of the 53 evaluable tumors showed overexpression of activated Akt which was associated with a poor prognosis: 1-year EFS was 59% for patients with Akt overexpression and 91% for those with no overexpression (*P* = 0.16); 1-year OS was 78% and 100%, respectively (*P* = 0.06) [55]. These data were confirmed in a further series of 32 pediatric GBM samples showing an association between Ras/Akt activation and poor survival [1]. In view of the frequency of Akt activation, the evaluation of molecularly targeted therapies that inhibit this pathway warrants consideration as far as these tumors are concerned.

## 6. Vascular Endothelial Growth Factor (VEGF)

HGGs are some of the most vascularised human tumours and the role of angiogenesis in malignant gliomas has been a very active area of research, with significant impact on the development of targeted therapy [56]. Vascular endothelial growth factor (VEGF) is an important regulator of angiogenesis which is strongly expressed in HGG (Figure 1). The degree of both vasculature density and VEGF expression is associated with the malignancy and aggressiveness of these tumors, as well as with outcomes such as clinical recurrence and survival [57–59]. Recently, data from clinical trials have established antiangiogenic therapy with VEGF targeted agents, with or without cytotoxic chemotherapy, as an active treatment option for patients with recurrent HGG who have failed previous TMZ therapy. Bevacizumab (BVZ) recently received Food and Drug Administration approval as a single agent for the treatment of patients with progressive GBM.

BVZ has been administered with irinotecan, a topoisomerase 1 inhibitor, in patients with recurrent HGG, and this combination has shown activity. BVZ in combination with irinotecan has now been reported in several trials to improve the outcome of recurrent malignant glioma. Both complete and partial responses, as well as disease stabilisation, have been described [60–62]. Moreover, recently, BVZ in combination with standard upfront treatment showed encouraging results [63].

Despite clear evidence of BVZ activity in recurrent HGG, not all patients respond to treatment, and no biomarkers for patients responsive to antiangiogenic therapies have been identified. Moreover, the activity of BVZ was found to be lower in pediatric gliomas, suggesting that genetic differences in pediatric gliomas might account for this difference.

A phase II pediatric brain tumor consortium study of a combination of BVZ and irinotecan was performed in children with recurrent malignant glioma by Gururangan et al., which reported a median PFS of 4.2 months and no sustained responses among the 15 children studied [64]. Similar results were reported in 12 children with recurrent or progressive HGG. Treatment tolerance and toxicity were comparable to adult HGG patients; however, the radiological response rate, response duration, and survival appeared inferior in pediatric patients [65]. Moreover bevacizumab was also investigated in combination with different drugs, such as irinotecan, CCNU, and TMZ, in children with recurrent or progressive WHO grades 3-4 gliomas, and although the combination was well tolerated, it lacked efficacy, with no sustained responses observed [66].

## 7. TP53

The* TP53* pathway is an important mechanism controlling the cell cycle (Figure 1). Activation of the tumor suppressor p53 by stress signals triggers different cellular programs such as cell cycle arrest, apoptosis, differentiation, DNA repair, autophagy, and senescence through complex network and signaling pathways. Childhood multi-institutional studies have confirmed that p53 overexpression and mutation appear to vary with age, being expressed more strongly in older versus younger children [67, 68]. In the Children's Cancer Group Study, the largest cohort of childhood HGGs analyzed to date,* TP53* mutations were observed in only 2/17 tumors (11.8%) from children <3 years of age at diagnosis versus 24/60 tumors (40%) from older children, a difference that is statistically significant (*P* = 0.04).

Moreover, a significant association has also been found between overexpression of p53, even in the absence of* TP53* mutations, and HGG outcome in children. The rate of PFS at 5 years was 44% in the group of patients whose tumors had low levels of expression of p53 and only 17% in the group of patients whose tumors had overexpression of p53 (*P* < 0.001) [68]. Moreover, overexpression of the p53 protein increases with tumor grade: one-fourth of analyzed AAs and half of GBMs overexpressed this protein.

## 8. Histone H3.3

Histones are eukaryotic nuclear proteins that play an important role in the regulation of DNA replication, transcription, and storage by changing the nucleosome structure depending on their post translational modifications. H3.3 is a replacement histone subclass that is encoded by 2 distinct genes,* H3.3A* (*H3F3A*) and* H3.3B* [69]. H3.3 is the major histone to be loaded on chromatin during brain development. This histone variant is known to modulate specific chromatin changes and gene expression profiles and to be associated with active chromatin and translation. Recent studies investigated the cancer genome of pediatric diffuse glioma [46, 70]. Exon sequencing identified mutations in histone H3.3 at either aminoacid 27, resulting in replacement of lysine by methionine (K27M), or at aminoacid 34, resulting in replacement of glycine by valine or arginine (G34V/R), as molecular drivers of a subgroup of pediatric and young adult GBMs and pediatric diffuse intrinsic pontine gliomas. In particular one-fourth of pediatric astrocytomas showed somatic mutations in* H3F3A* gene [46, 70–73].* G34V/R-H3.3* was mainly seen in older patients (median age 20 years) and almost exclusively in hemispheric HGGs [53]. Notably, in GBM patients G34 mutant also showed a trend toward a better OS than wild-type tumor patients, with marginal statistical significance (*P* = 0.05) [55]. In contrast, patients who harbored the* K27M-H3.3* mutation tended toward a worse overall survival when compared to patients who were wild-type for* H3.3*. Moreover,* H3F3A K27* mutation appeared to be exclusive to pediatric high-grade gliomas (median age 11 years) [46, 73] and it was prevalent in diffuse intrinsic pontine glioma [70, 72]. Therefore this mutation defines clinically and biologically distinct subgroup and it is suitable as a molecular marker for pediatric diffuse high-grade astrocytomas with a future impact on therapeutic trial design.

## 9. Alpha-Thalassemia/Mental-Retardation Syndrome X-Linked (ATRX)

The* ATRX* (alpha-thalassemia/mental-retardation syndrome X-linked) gene is located on chromosome Xq21.1 and encodes a subunit of a chromatin remodelling complex required for H3.3 incorporation at pericentric heterochromatin and telomeres [74]. Mutations that inactivate* ATRX* gene are common in human pancreatic neuroendocrine tumors and central nervous system tumors [70–75]. Loss of ATRX function impairs the heterochromatic state of the telomeres, perhaps because of reduced incorporation of chromatin onto H3.3 histones [75]. This leads to telomere destabilization, which results in a telomerase-independent telomere maintenance mechanism called alternative lengthening of telomeres (ALT).

In 2011, mutations in the* ATRX* gene were described for the first time in a small fraction of adult and pediatric GBM, as well as oligodendrogliomas, and a significant correlation with ALT was demonstrated [75]. Recently, mutations and loss of ATRX have been reported in one-third of pediatric GBMs [46] and 7% of adult GBMs [75]. In gliomas,* ATRX* mutation has been associated with a better prognosis in anaplastic gliomas [76].

Khuong-Quang et al. showed that the presence of* ATRX* mutation significantly overlapped with* TP53* mutations in GBM (*P* = 0.01) regardless of the location within the brain and with G34V/R mutants in supratentorial GBM (*P* < 0.0001). Moreover* ATRX* mutations were infrequent in DIPG and mainly occurred in older children (*P* < 0.0001). The low incidence of this mutation in DIPG could be an age-related phenomenon as the mean age of DIPG cohort was 7 versus 12 years for the supratentorial GBM patient cohort [72]. The requirement for* ATRX* mutations in GBM may thus be due to tumor location and/or the age of the patient. This is potentially indicative of a different cell of origin or age-related plasticity of the tumor.

## 10. Isocitrate Dehydrogenase (IDH)

Recently a high frequency of mutations of the isocitrate dehydrogenase (*IDH1* and* IDH2*) genes, which encode the IDH enzymes, was detected in adult secondary GBM (85%). These alterations inhibit the normal function of the IDH enzyme in converting isocitrate to *α*-ketoglutarate [77] and instead drive the conversion of *α*-ketoglutarate to R(-)-2-hydroxyglutarate [35], a metabolite that may contribute to tumor development.* IDH* mutations likely represent an early step in tumorigenesis because such alterations are also observed commonly in preexisting lower grade lesions [78].

Such alterations are uncommon in pediatric population, highlighting molecular differences with adult secondary GBM.

The series of Balss et al. [79] included 14 pediatric GBMs and only one case had an* IDH1* mutation. In 2 recent studies performed on pediatric malignant gliomas* IDH1* mutations were not detected in any case [80, 81]. Similarly, in a study of 1010 diffuse gliomas that included a small subgroup of children, Hartmann et al. [82] noted that* IDH1* mutations were rare in the pediatric subset, and* IDH2* mutations were absent, although the frequency of mutations as a function of age and histology was not provided.

Based on the molecular similarities that have been noted between primary pediatric malignant gliomas that arise in older children and secondary malignant gliomas that occur in adults, recently Pollack et al. examined the frequency of* IDH* mutations in a cohort of 43 HGG patients aged 3–21 years at the time of diagnosis [83].* IDH1* mutations were noted in 7 of 20 tumors (35%) from children ≥14 years, but in 0 of 23 (0%) younger children (*P* = 0.01), suggesting that a subset of such lesions may be comparable on a molecular basis to lesions that arise in young adults. In contrast, such alterations were rare in tumors arising in younger children, supporting the existence of age-related pathways of tumorigenesis in childhood. Moreover* IDH*-mutated tumors seemed to be associated with a more favorable prognosis than non mutated tumors as in adult patients [80].

## 11. Conclusion

HGGs are highly heterogeneous and aggressive brain tumours, requiring a multidisciplinary approach. Recently, molecular research has significantly improved our understanding of glioma pathogenesis and identified key proteins that regulate normal biological processes and cellular pathways associated with pediatric HGG. The identification of agents directed against molecular targets aims at going beyond the traditional therapeutic approach in order to develop a personalized therapy and improve the outcome of pediatric HGG.

Results from adult clinical trials cannot simply be extrapolated to children due to crucial molecular differences between adult and pediatric HGG, drawing attention to the need for exclusively pediatric clinical studies. PDGFRA is the most frequent target of focal amplification in pediatric HGGs; in contrast, EGFR is the predominant receptor tyrosine kinase targeted in adult GBM. No* IDH1* mutations have been found in pediatric tumors, highlighting molecular differences with adult secondary GBM. Activity of bevacizumab is lower in pediatric gliomas than that observed in adult HGG. Recently a central role of* ATRX* and* H3F3A K27* mutations in pediatric HHGs has been described.

Although targeted agents offer great promise, to date the overall response rate has been disappointing, with differences in response not only between children and adults but also within each population. These observations demonstrate that distinct biological profiles can be identified in different subsets of patients; therefore individual targeted therapy based on molecular biology should be investigated in order to define an optimal treatment strategy for each patient.

## Figures and Tables

**Table 1:** Summary of the main clinical trials in pediatric HGG.

Agent and mechanism of action	Treatment	Study design	Status at diagnosis	Outcome	Reference
Alkylating agent (TMZ)	RT + TMZ	Phase II	Newly diagnosed HGG	EFS3y: 11 ± 3% OS3y: 22 ± 5%	[4]
	TMZ	Phase II	Relapsed or progressive HGG	mOS 4.7 months response rate: 12%	[5]
	RT + TMZ	Phase II	Newly diagnosed HGG	PFS1y: 43% ± 9% PFS2y: 11% ± 5% OS1y: 63% ± 8% OS2y: 21% ± 7%	[6]
	TMZ	Phase II	Relapsed or progressive HGG	mPFS: 3 months PFS6m: 33% mOS: 4 months OS6m: 37.5%	[7]

Receptor tyrosine kinase inhibitors					
EGFR inhibitor (erlotinib)	RT + erlotinib	Phase I	Newly diagnosed HGG	PFS1y: 56% ± 10% PFS2y: 35% ± 12% OS1y:78% ± 9% OS2y: 48% ± 12%	[31]
PDGFR inhibitor (imatinib)	Erlotinib	Phase I	Relapsed or progressive HGG	mPFS: 1.5 months mOS: 4.1 months SD: 28%	[33]
	Imatinib	Phase I	Relapsed or progressive HGG	EFS6m: 17.9% ± 6.6% EFS12m: 0%	[51]

Antiangiogenic agent (BVZ)	BVZ + irinotecan	Phase II	Relapsed or progressive HGG	mPFS: 2.25 months mOS: 6.25 months SD: 33.3%	[65]
	BVZ + irinotecan	Phase II	Relapsed or progressive HGG	mPFS: 4,2 months PFS6m: 41.8%	[64]
	BVZ + irinotecan + TMZ (6 patients) BVZ + irinotecan (1 patient) BVZ + CCNU (1 patient)	Phase II	Relapsed or progressive HGG	mPFS: 15 weeks mOS: 30 weeks	[66]

TMZ: temozolomide; mOS: median overall survival; OS1y: overall survival at 1 year; OS2y: overall survival at 2 years; OS3y: overall survival at 3 years; mPFS: median progression-free survival; PFS1y: progression-free survival at 1 year; PFS2y: progression-free survival at 2 years; SD: stable disease; EFS6m: event-free survival at 6 months; EFS12m: event-free survival at 12 months; EFS3y: event-free survival at 3 years; RT: radiotherapy; CT: chemotherapy; BVZ: bevacizumab; GBM: glioblastoma multiforme.
